# The coordination between root and leaf functional traits across 33 woody plant species shifts between mycorrhizal types

**DOI:** 10.1093/treephys/tpaf151

**Published:** 2025-12-10

**Authors:** Katsumi C Suzuki, Hirofumi Kajino, Shusaku Hirokawa, Hajime Tomimatsu, Kohmei Kadowaki, Kouki Hikosaka

**Affiliations:** Graduate School of Life Science, Tohoku University, Aramakiaza Aoba 6-3, Aoba-ku, Sendai, Miyagi 980-8578, Japan; Graduate School of Life Science, Tohoku University, Aramakiaza Aoba 6-3, Aoba-ku, Sendai, Miyagi 980-8578, Japan; Graduate School of Life Science, Tohoku University, Aramakiaza Aoba 6-3, Aoba-ku, Sendai, Miyagi 980-8578, Japan; Graduate School of Life Science, Tohoku University, Aramakiaza Aoba 6-3, Aoba-ku, Sendai, Miyagi 980-8578, Japan; The Hakubi Center for Advanced Research, Kyoto University, Yoshida-honmachi, Sakyo-ku, Kyoto 606-8501, Japan; Graduate School of Agriculture, Kyoto University, Kitashirakawa Oiwake-cho, Sakyo-ku, Kyoto 606-8502, Japan; Graduate School of Life Science, Tohoku University, Aramakiaza Aoba 6-3, Aoba-ku, Sendai, Miyagi 980-8578, Japan

**Keywords:** arbuscular mycorrhizal fungi, ectomycorrhizal fungi, plant economics spectrum, specific leaf area, specific root length

## Abstract

Root and leaf traits are expected to converge on the plant economics spectrum (PES). Some studies have focused on correlation between specific root length (SRL) and specific leaf area (SLA), which reflect resource acquisition per invested mass in root and leaf, respectively. However, the results have been inconsistent amongst previous studies. We hypothesized that this discrepancy was due to overlooked variations in root traits depending on mycorrhizal types because SRL can be influenced by not only PES but also mycorrhizal types. To assess how mycorrhizal type inherently mediates the coordination of root and leaf traits, we determined the leaf and root traits of current-year seedlings of 33 species encompassing different leaf habits and mycorrhizal types, AM (arbuscular mycorrhizal) and ECM (ectomycorrhizal) species, grown under a common condition. Root and leaf traits correlated with the first axis of the principal component analysis, and this axis represented PES. Root diameter (RD) also correlated with the second axis, which differed between mycorrhizal types. Specific root length (SRL) and SLA were correlated positively to each other, but ECM species had higher SRL than AM species when compared at the same SLA. This was because (i) SRL is negatively related to root tissue density (RTD) and RD, (ii) RTD was negatively correlated with SLA and (iii) RD was smaller in ECM. Leaf and root traits are tightly coordinated with each other across species, but the relationship shifts between the mycorrhizal types.

## Introduction

Plant strategy varies along an axis running from resource acquisitive to resource conservative species as a result of resource allocation (Reich et al. 1992, Wright et al. 2004, Díaz et al. 2016). Resource acquisitive species have higher specific leaf area (SLA; leaf area per leaf dry mass), leaf nitrogen concentration (LN) and photosynthetic rates, with a shorter leaf life span and lower leaf toughness than resource conservative species (Wright et al. 2004, Onoda et al. 2011). This leaf trait variation alongside resource acquisitive to conservative strategy is called the leaf economics spectrum (LES) (Reich et al. 1992, Wright et al. 2004).

Root traits such as specific root length (SRL; root length per root mass), root tissue density (RTD; root mass per volume) and root nitrogen concentration (RN) have also been considered to vary along the economics spectrum. Earlier studies have indicated that root traits converge on an axis like leaf traits (root economics spectrum) (Comas and Eissenstat 2004, Roumet et al. 2016, de la Riva et al. 2021*a*). However, later studies have reported that root trait variation can be explained by two major axes: the economics spectrum from resource acquisitive to conservative strategy and dependency on mycorrhizal symbiosis for nutrient uptake (Kramer-Walter et al. 2016, McCormack and Iversen 2019, Bergmann et al. 2020, Weigelt et al. 2021, Da et al. 2023, Ding et al. 2023). These axes are called the conservation gradient and collaboration gradient, respectively, and compose the root economics space (Bergmann et al. 2020). The latter axis represents plant nutrient acquisition strategies: from species highly relying on mycorrhizal symbiosis for nutrient uptake (‘outsourcing’) to species mainly absorbing nutrients through their own roots (‘do-it-yourself’). ‘Outsourcing’ species have larger root diameter (RD) and lower SRL than ‘do-it-yourself’ species (Bergmann et al. 2020).

Root traits also change depending on mycorrhizal types. Species colonized by arbuscular mycorrhizal fungi (AM) have larger RD than species colonized by ectomycorrhizal fungi (ECM) because AM species have thick root cortex to colonize their fungal partners (Brundrett 2002, Comas et al. 2014, Ma et al. 2018, Yan et al. 2022). Thick root cortex is needed for symbiosis with AM fungi that colonizes in the root cell with arbuscules or vesicles (Brundrett 2002, Ma et al. 2018). In contrast, ECM fungi colonized root surface or intercellular space; thus ECM species do not need thick root cortex and their root is thinner than AM species (Brundrett 2002, Comas et al. 2014, Ma et al. 2018, Yan et al. 2022).

Because leaf and root traits are associated with the uptake rate of carbon and other nutrients, respectively, it has been expected that root traits are coordinated with leaf traits across species on an axis, called the plant economic spectrum (PES) (Westoby and Wright 2006, Freschet et al. 2010, Reich 2014). Higher root nitrogen acquisition abilities are essential in keeping leaf nitrogen concentration higher in species with higher carbon gain to hold positive correlation between photosynthesis rate and LN (Hikosaka and Osone 2009). Experimental studies have demonstrated significant correlations between root and leaf traits, e.g., RN vs LN and RTD vs leaf tissue density, as expected (Craine and Lee 2003, Freschet et al. 2010, de la Riva et al. 2021*b*, Mueller et al. 2024). However, the relationship between SRL and SLA, both of which represent resource acquisition per invested mass (Eissenstat 2002, Reich 2014), was not straight forward and the conclusions were not necessarily consistent amongst studies (Weemstra et al. 2016, Weigelt et al. 2021); positive correlation between SRL and SLA has been observed in some previous studies (Holdaway et al. 2011, de la Riva et al. 2021*b*, Mueller et al. 2024), but not in other cases probably due to different natural selection between leaf and root trait (Chen et al. 2013, Valverde-Barrantes et al. 2015, Burton et al. 2020).

A primary reason for the apparent discrepancy amongst previous studies on the relationship between root and leaf traits might be that they have not fully considered the effects of mycorrhizal types on root traits. We can expect that SRL is related not only with economics spectrum but also with mycorrhizal types. Specific root length (SRL) is expressed as a function of RD and RTD (Ostonen et al. 2007):


(1) \begin{equation*} \mathrm{SRL}=\frac{4}{\mathrm{\pi} \times \mathrm{R}{\mathrm{D}}^2\times \mathrm{R}\mathrm{TD}} \end{equation*}

The root economics space concept suggest that RTD is related to the economics spectrum, whereas RD is related to the collaboration gradient and/or mycorrhizal types as mentioned above. Therefore, SRL, which is inversely proportional to RD squared and RTD, should reflect not only the economics spectrum but also the root collaboration gradient. Differences in SRL depending on mycorrhizal type can obscure the positive correlation between SRL and SLA. However, previous studies have not examined how the coordination between root and leaf traits was influenced by the specific types of mycorrhizal symbiosis associated with plant species.

Furthermore, we note that five additional factors might contribute to the discrepancy in trait coordination. First, trait plastic change with environmental heterogeneity may affect the relationship between root and leaf traits. Many studies have assessed traits of root and leaf sampled in the field. However, root and leaf traits change even within species depending on environmental factors such as resource availability, and the degree of intraspecific variations in trait values differs amongst species (Weemstra et al. 2017, 2021, Wang et al. 2018). In particular, SRL and SLA change plastically with light conditions and soil nutrients, which can complicate their relationship (Freschet et al. 2015). Second, ontogeny also influences root and leaf traits. For example, younger or smaller individuals tend to have larger SLA than adults (He and Yan 2018, Liu et al. 2020, Lusk and Warton 2007, Tomas and Winner 2002). Plant size is often correlated with RTD and SRL, and smaller individuals have more resource acquisitive roots (Garbowski et al. 2021, Leroy et al. 2023). Third, intraspecific variation in root traits may arise from plastic responses to mycorrhizal infection (Kilpeläinen et al. 2019, McCormack and Iversen 2019), though the results were different amongst studies; Romero et al. (2023) reported that individuals inoculated with AM have larger RD and smaller SRL compared with those without mycorrhizal infection, whilst Kilpeläinen et al. (2019) reported the opposite trend. These results imply that even within the same species, there is a difference in infection levels, affecting root traits and thus root–leaf trait relationships, which likely occurs if field-grown plants are examined. Fourth, biomass allocation between leaf and root can regulate their functional balance; lower root activity per root mass can be offset by greater biomass allocation to roots (Weemstra et al. 2020). Differences in biomass allocation to roots may influence relationship between SRL and SLA. Fifth, phylogeny influences trait–trait relationships (Kong et al. 2014, Bergmann et al. 2017, Valverde-Barrantes et al. 2017). Relationship between SRL and SLA changes depending on the clades: positive correlation is observed in Fabids, monocots and Caryophyllales, but gymnosperms, asterids and magnoliids do not show significant correlation (Valverde-Barrantes et al. 2017).

In this study, we explored how root and leaf traits are inherently correlated with each other across species associated with different types of mycorrhizal symbiosis. We conducted a common garden experiment of 33 dominant woody species in Japan, including different leaf habits (evergreen conifer, evergreen broadleaf and deciduous broadleaf trees) and mycorrhizal types (species symbiotic with AM and ECM) and compared root and leaf traits. To focus on inherent differences amongst species, we used current-year seedlings grown in a common environment to minimize potential intraspecific variation driven by environmental heterogeneity, ontogeny or mycorrhizal infection. We tested the hypothesis that root and leaf traits are correlated with each other, but the relationship is affected by the mycorrhizal types. We also studied biomass allocation between aboveground and belowground parts. In addition, we applied phylogenetic independent contrast method to examine the effect of phylogenetic constraint.

## Materials and methods

### Species selection and growth conditions

We selected woody species that are abundant in the typical natural forests in Japan based on the tree census data from the Monitoring Sites 1000 Project by the Ministry of the Environment, Japan (Ishihara et al. 2011). We selected 33 woody species (Table S1 available as Supplementary Data at *Tree Physiology* Online) whose average basal area in each forest plot was larger than 1.5 m^2^ per ha or which occurred in more than three sites in the 25 forest sites from Uryu (44°22′11′′ N, 142°16′48′′ E) to Yakushima (30°22′12′′ N, 130°23′24′′ E) in the Monitoring Sites 1000 Project, and we excluded liana species. To obtain a wide variation in leaf and root traits, the 33 species included 19 deciduous broadleaf, 7 evergreen broadleaf and 7 evergreen coniferous species. The mycorrhizal types of each species were classified based on the literature (Maherali et al. 2016, Soudzilovskaia et al. 2020). Because 26 species lacked species-level symbiotic mycorrhizal type in the references, the most dominant mycorrhizal type within the genus was selected as the mycorrhizal type for those species. The species number of each mycorrhizal type was 16 AM, 16 ECM and 1 ericoid mycorrhizal (ERM) species. All species in our experiment were regarded as obligately mycorrhizal species in these databases. We collected seeds of the selected woody species from the tree garden of Forestry and Forest Products Research Institute (Tsukuba, Ibaraki, Japan), Ashiu Forest Research Station, Kyoto University (Nantan, Kyoto, Japan) and the seed bank of Forest Tree Breeding Center (Hitachi, Ibaraki, Japan). Plants were grown in an experimental outdoor garden of the Faculty of Science, Tohoku University in Sendai, Japan (38°15′15′′ N, 140°50′50′′ E, 139 m above sea level). The mean annual temperature was 12.8 °C, and the annual precipitation was 1276.7 mm during 1991–2020 (Japan Meteorological Agency). We used New Wagner Pot (Upper diameter, lower diameter and height were 174.6, 160.4 and 197.5 mm, AS ONE Corporation, Osaka, Japan) filled with washed river sand to cultivate seedlings. We did not use any soil and inoculation because we were interested in genetic variations in functional traits amongst species and tried to avoid phenotypic changes caused by mycorrhizal infection. Seeds were sown in pots filled with the sand at the end of May 2022, and the germinated plants were replanted in pots filled with river sand (one individual per pot). The maximum number of individuals per species was 20. They were watered twice a week and fertilized from germination to sampling with liquid fertilizer (HYPONeX NPK 6–10-5 Hyponex Japan, Osaka, Japan) diluted with tap water, which contains nitrogen, phosphorus and potassium with trace elements, once a week to supply 3 mg of nitrogen per plant.

### Sampling and trait determination

We sampled whole seedlings of three to six individuals per species from 2 September to 6 October in 2022. The age of harvested seedlings varied from 2 to 3 months old because of variation in the germination time. Above- and belowground parts of the plant were defined as the plant body emerging from the pot and buried in sand, respectively. Fine roots were defined as first- and second-order roots and sampled according to Freschet et al. (2021). We washed roots with water and cut two undamaged fine root branch networks composed solely of first- and second-order roots from each individual. Each fine root was scanned with 800 d.p.i. using a flatbed scanner (CanoScan LiDE 210, Canon, Tokyo). We measured scanned root length and diameter and estimated its volume and surface area using Smartroot in ImageJ PlugIn (Lobet et al. 2011). We scanned all leaves of sampled individuals with 300 dpi using the scanner (CanoScan LiDE 210, Canon, Tokyo). Leaf area was obtained by measuring the area of scanned leaf images with ImageJ. Leaf force to punch (LFP) was measured as indicator for leaf toughness using a penetrometer with a metal cylinder of 2 mm in diameter attached to a load sensor (DS2-50 N, IMADA, Toyohashi) according to Onoda et al. (2011). The stem volume was estimated from the length and diameter of the base, centre and apex of the stem by assuming two circular truncated cones because their stem were too small to measure the volume by the water displacement method (Pérez-Harguindeguy et al. 2013). Leaves, stems and whole and fine roots were dried at 70 °C in an oven for ˃3 days before being weighed.

Root diameter (RD) was obtained by calculating the average of the diameters of the scanned fine roots. Specific root length (SRL) and specific root area (SRA) were calculated as the total length and total surface area of the root per dry mass. Root tissue density (RTD) was calculated as dry mass divided by the volume of the roots. Fraction of first-order root length (mm mm^−1^) was calculated as total first order root length divided by total fine root length. Specific leaf area (SLA) and stem tissue density (STD) were obtained as leaf area per leaf dry mass and stem dry mass per stem volume, respectively. Fine root nitrogen concentration (RN) and leaf nitrogen concentration (LN) were determined with an elemental analyzer (UNICUBE, Elementar, Germany). Leaf force to punch (LFP) was calculated as the maximum force applied to the cylinder during penetration divided by the circumference of the cylinder based on the method of Onoda et al. (2011). Above- and belowground mass ratio (ABR) was calculated by dividing the sum of leaf and stem mass by root mass. We confirmed that ECM species had denser root branches and smaller root diameters than AM species, which is consistent with a previous study (Comas et al. 2014) (Figure S1 available as Supplementary Data at *Tree Physiology* Online). The growth form of studied species was classified based on Hayashi (2014). Selected species included 3 shrub and 30 tree species. We did not perform any artificial inoculation to the studied plants. We checked ECM infection by observing scanned root visually, and ECM infection was not considered to occur in our experiment. We did not check whether AM infection occurred or not.

### Statistical analysis

All trait values were log-transformed (log_10_) and analyzed using R 4.1.1. We performed two principal component analysis (PCA) for species trait mean. One is the Standard PCA using *prcomp*. The other is the Phylogenetic PCA (Revell 2009) using *phyl.pca* in the R package ‘phytools’ (Revell 2012) to test how phylogeny influences the trait–trait relationships because root traits are known to be strongly influenced by phylogeny (Kong et al. 2014, Bergmann et al. 2017, Valverde-Barrantes et al. 2017). We calculated Pagel’s λ (Pagel 1999), where the higher value indicates a stronger phylogenetic signal in the trait, using *phylosig* on the R package ‘phytools’. Phylogenetic independent contrasts (PICs) value of each trait at internal nodes in phylogenetic tree was calculated using *pic* in the R package ‘ape’ (Paradis et al. 2004). We tested phylogenetic effects on trait–trait correlation by evaluating the correlation between PICs of traits at all internal nodes based on Harvey and Pagel (1991). Phylogenetic tree (Figure S2 available as Supplementary Data at *Tree Physiology* Online) was created based on phylogenetic information available and using *phylo.maker* on the R package ‘V. phyloMaker2’ (Jin and Qian 2022). Standard major axis (SMA) regression was performed using *sma* on the R package ‘smatr’ (Warton et al. 2012) to test the Standard correlation between the traits using species means. SMA uses the type 2 regression, which is more appropriate than the type 1 regression when both variables were measured traits and might have errors, and neither variable is considered the predictor (Warton et al. 2006). We compared the slopes and intercepts of the regression lines between mycorrhizal types and leaf habits, also using the *smatr* package in R (Warton et al. 2012). We conducted two-way ANOVA with *lm* and *Anova* on the R package ‘car’ (Fox and Weisberg 2019) to check how leaf habit and mycorrhizal types affected traits. In the two-way ANOVA, traits were used as the objective variable, and leaf habits and mycorrhizal type were used as the explanatory variables. We excluded *Pieris japonica* from the two-way ANOVA and the comparisons of regression slopes and intercepts between mycorrhizal types and leaf habits because it was the only species associated with ericoid mycorrhizal fungi in our dataset. Statistical significance was set at *P* < 0.05.

## Results

**Figure 1:**
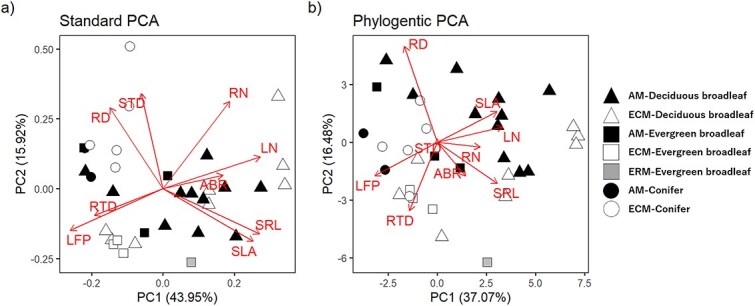
The result of Standard PCA (a) and Phylogenetic PCA (b) coded by mycorrhizal types and leaf habit. Specific root area (SRA) was excluded because specific root length (SRL) and SRA were strongly correlated. In Phylogenetic PCA, PC1 axis was flipped by multiplying PC1 score for each trait and species by −1. RD, root diameter; SRL, specific root length; RTD, root tissue density; RN, root nitrogen concentration; ABR, above- and belowground ratio; SLA, specific leaf area; LFP, leaf force to punch; LN, leaf nitrogen concentration; AM, species symbiotic with arbuscular mycorrhizal fungi; ECM, species symbiotic with ectomycorrhizal fungi; ERM, species symbiotic with ericoid mycorrhizal fungi.

Trait values varied amongst species, and the coefficient of variation of each trait was from 24.4% in RN to 98.1% in SRL (Table S2 available as Supplementary Data at *Tree Physiology* Online). In the Standard PCA (Figure 1a), PC1 represented 49.12% of the total variation and was significantly correlated with all the studied traits (*P* < 0.05), and PC2 significantly correlated with RD, SRL, RN and LFP (*P* < 0.05) (Table 1). Pagel’s λ showed a phylogenetic signal for some traits (Table S2 available as Supplementary Data at *Tree Physiology* Online). Conifer species had extreme values in PIC; PIC of RN was highest at the node of the genus *Picea* (Figure S3 available as Supplementary Data at *Tree Physiology* Online) and PIC of RTD was lowest at the node of the genus *Abies* and highest at the node of the genus *Picea* (Figure S4 available as Supplementary Data at *Tree Physiology* Online). However, the result of the Phylogenetic PCA (Figure 1b) was basically similar to that of the Standard PCA. All the studied traits significantly correlated with PC1 in both the Standard PCA (R^2^ = 0.27–0.72, *P* < 0.01) and Phylogenetic PCA (R^2^ = 0.22–0.74, *P* < 0.01). Root diameter (RD) was strongly correlated with PC2 in both the Standard PCA (R^2^ = 0.68, *P* < 0.001) and Phylogenetic PCA (R^2^ = 0.64, *P* < 0.001). Exceptionally, there were different results in significance between the Standard and Phylogenetic PCA in three traits: SRL and RN, which were significantly correlated with PC2 in the Standard PCA (*P* < 0.05), were not correlated with PC2 in the Phylogenetic PCA, whereas RTD was not significantly correlated with PC2 in the Standard PCA, but in the Phylogenetic PCA (*P* < 0.05).

**Table 1:** Pearson coefficients between PC1 and PC2 in the standard PCA and the phylogenetic PCA (Figure 1) and traits, and proportion of variance in the standard PCA and the phylogenetic PCA (Figure 1). Specific root area (SRA) was excluded because specific root length (SRL) and SRA were strongly correlated. In standard PCA, PC2 axis was flipped by multiplying PC2 score for each trait and species by −1. RD, root diameter; SRL, specific root length; RTD, root tissue density; RN, root nitrogen concentration; ABR, above- and belowground ratio; SLA, specific leaf area; LFP, leaf force to punch; LN, leaf nitrogen concentration.

	Standard PCA		**Phylogenetic PCA**	
	PC1	PC2	PC1	PC2
**RD**	−0.46^**^	0.83^***^	−0.51^**^	0.80^***^
**SRL**	0.85^***^	−0.46^**^	0.86^***^	−0.33
**RTD**	−0.60^***^	−0.26	−0.56^***^	−0.49^**^
**RN**	0.60^***^	0.39^*^	0.56^***^	0.03
**ABR**	0.52^**^	0.22	0.47^**^	−0.12
**SLA**	0.77^***^	−0.17	0.82^***^	0.19
**LFP**	−0.82^***^	−0.36^*^	−0.81^***^	−0.37^*^
**LN**	0.86^***^	0.12	0.85^***^	0.13
**Proportion of variance (%)**	49.12	16.76	41.18	18.64

^***^ *P* < 0.001, ^**^P < 0.01, ^*^P < 0.05.

**Figure 2:**
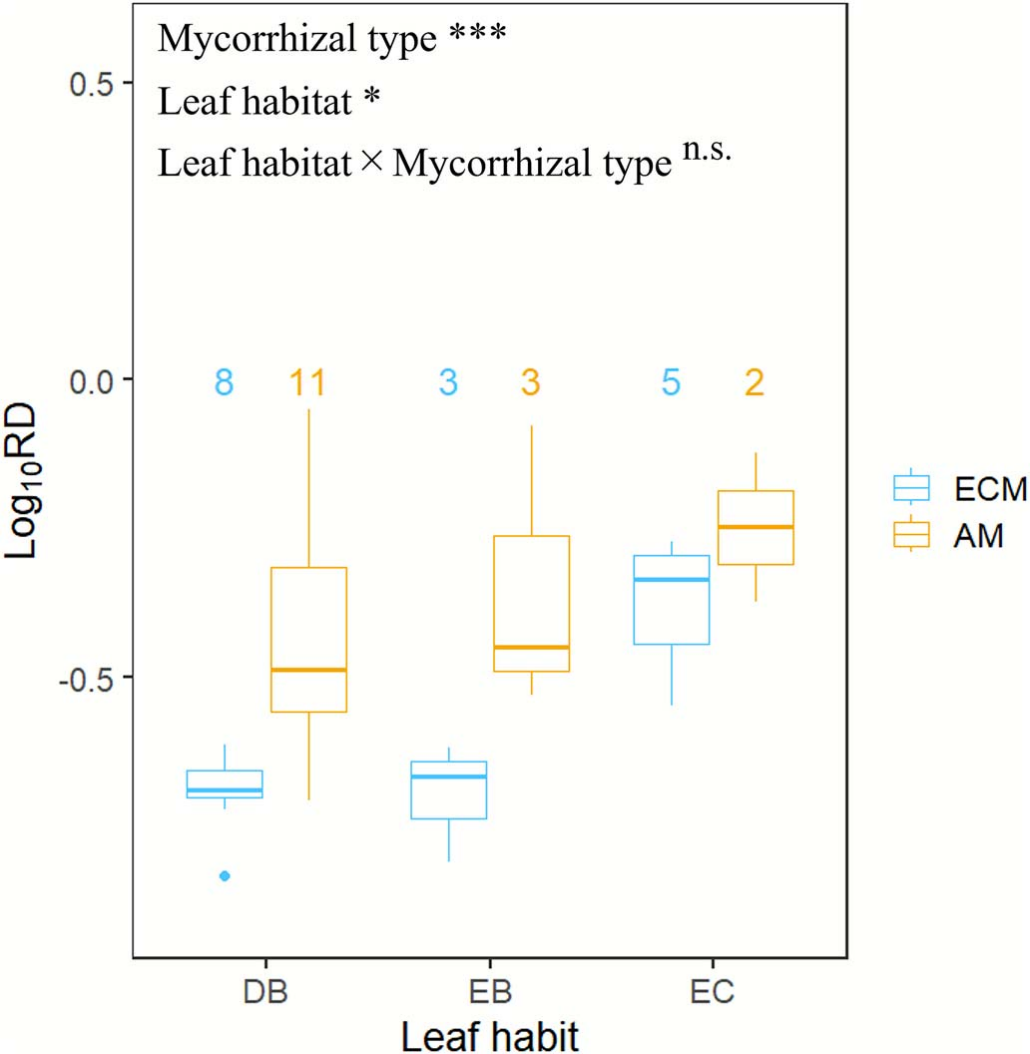
Comparison of root diameter (RD, mm) amongst mycorrhizal types (AM, species symbiotic with arbuscular mycorrhizal fungi; ECM, species symbiotic with ectomycorrhizal fungi) and leaf habit (DB, deciduous broadleaf; EB, evergreen broadleaf; EC, evergreen conifer). Result of two-way ANOVA is shown in upper (^*^P < 0.05, ^**^P < 0.01, ^***^P < 0.001), and the effects are listed in order: Mycorrhizal type (main effect), leaf habit (main effect) and leaf habit × mycorrhizal type (interaction). The number above box shows the number of species behind each boxplot.

PC1 score in both the Standard and Phylogenetic PCA was significantly different amongst leaf habits (*P* < 0.01) (Figure S5a and c available as Supplementary Data at *Tree Physiology* Online). PC2 score varied by leaf habit only in the Standard PCA (Figure S5b and d available as Supplementary Data at *Tree Physiology* Online), but differed by mycorrhizal types in both the Standard and Phylogenetic PCA (*P* < 0.01) (Figure S5c and d available as Supplementary Data at *Tree Physiology* Online). The interaction between leaf habits and mycorrhizal types had no significant effect on the four axes. AM species had larger RD than ECM species across leaf habit. Root diameter (RD) was influenced by mycorrhizal type and leaf habit, but their interaction effect on RD was not significant (Figure 2). Specific root length (SRL) was also altered by mycorrhizal types and leaf habit. Mycorrhizal types did not influence RTD, ABR and RN and fraction of first-order root length, whilst RTD was affected by leaf habit (Figure S6 available as Supplementary Data at *Tree Physiology* Online).

Traits that correlated with PC1 in the Standard and Phylogenetic PCA were correlated with each other (*P* < 0.05) (Table 2). Specific root length (SRL), SLA and LN were significantly correlated with RD that correlated with PC2 in the Standard and Phylogenetic PCA (*P* < 0.05). The PICs of SRL and SLA were not significantly correlated with each other due to outlier at the node of genus *Picea* (Figure S7 available as Supplementary Data at *Tree Physiology* Online) and conifer species. When PIC value at the node of genus *Picea* was excluded, this relationship was marginally positive (R^2^ = 0.11, *P* = 0.06) (Figure S7 available as Supplementary Data at *Tree Physiology* Online). When PIC values at node conifer were excluded, the relationship was significantly positive (R^2^ = 0.15, *P* < 0.05) (Figure S7 available as Supplementary Data at *Tree Physiology* Online). Other relationships (e.g., LN-RN, SRL-RN, LN-SLA) were also changed when they were evaluated using PICs (Table 2).

**Table 2:** Correlation coefficients between trats. Upper right of this table shows coefficients of the standard correlation and lower left of this table shows coefficients of the PICs correlation. RD, root diameter; SRL, specific root length; SRA, specific root area; RTD, root tissue density; RN, root nitrogen concentration; STD, stem tissue density; ABR, above- and belowground ratio; SLA, specific leaf area; LFP, leaf force to punch; LN, leaf nitrogen concentration.

	**RD**	**SRL**	**SRA**	**RTD**	**RN**	**STD**	**ABR**	**SLA**	**LFP**	**LN**
**RD**		−0.76^**^	−0.44^**^	−0.16	−0.11	−0.07	−0.05	−0.36^*^	−0.07	−0.15
**SRL**	−0.46^**^		0.91^***^	−0.53^**^	0.33	0.22	0.37^*^	0.67^***^	−0.45^**^	0.59^***^
**SRA**	0.11	0.87^***^		−0.80^***^	0.38^*^	−0.16	0.39^*^	0.65^***^	−0.51^**^	0.59^***^
**RTD**	−0.62^***^	0.31^*^	0		−0. 28	0.19	−0.21	−0.48^**^	0.41^*^	−0.37^*^
**RN**	0.35^*^	0.63^***^	0.59^***^	0.33		0.12	0.38^*^	0.18	−0.51^**^	0.53^**^
**STD**	0.02	0.70^***^	0.83^***^	0.43^*^	0.68^***^		−0.26	−0.52^**^	0.23	−0.10
**ABR**	0.26	0.77^**^	0.72^***^	0.22	0.61^***^	0.73^***^		−0.27	0.42^*^	−0.28
**SLA**	−0.04	−0.13	−0.22	0.39^*^	−0.50^**^	−0.51^**^	0.20		−0.64^***^	0.52^**^
**LFP**	0.40^*^	−0.05	0	−0.26	0.16	−0.01	−0.02	−0.53^***^		−0.79^***^
**LN**	−0.64^***^	−0.24	−0.51^**^	0.38^*^	−0.02	−0.31	0.39^*^	0.12	−0.40^*^	

^***^ *P* < 0.001, ^**^P < 0.01, ^*^P < 0.05.

**Figure 3:**
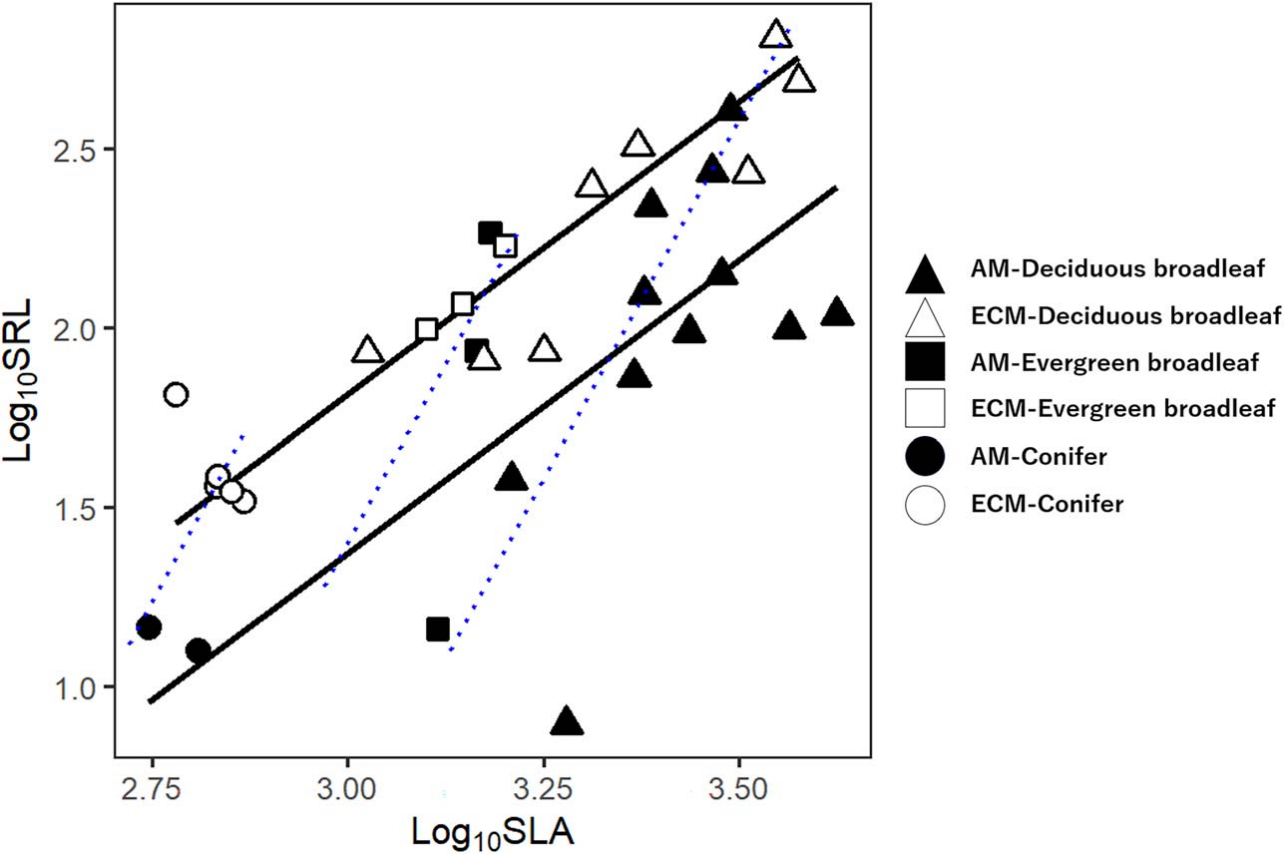
The log–log relationship between specific root length (SRL, m g^−1^) and specific leaf area (SLA, cm^2^ g^−1^) in leaf habits and mycorrhizal types. Blue lines show regression for each leaf habit at the common slope, and dotted line indicates weak or non-significance correlation (deciduous broadleaf species, y = 4.00x − 11.5: R^2^ = 0.26, *P* < 0.05; evergreen broadleaf species, y = 4.00x − 10.7: R^2^ = 0.40, *P* = 0.18; evergreen conifers, y = 4.00x − 9.81: R^2^ = 0.1, *P* = 0.49). Black lines show regression for each mycorrhizal type at the common slope (see Table 3 for R^2^, significances and equations).

Specific root length (SRL) had a significantly positive correlation with SLA in the Standard correlation across the studied species (R^2^ = 0.45, *P* < 0.001) (Table 2). When the correlation was separately applied to AM and ECM species (Figure 3), the correlation in two mycorrhizal types was stronger than that using all species (Table 3). To assess the effect of phylogenetic distance on the relationship between SRL and SLA for each mycorrhizal type, we analyzed data that excluded conifers. The SRL—SLA relationship was also strong in two mycorrhizal types (Table S3 available as Supplementary Data at *Tree Physiology* Online). When the analysis was applied to different leaf habit species across different mycorrhizal types (Figure 3), the correlation was significantly positive in deciduous broadleaf species (R^2^ = 0.26, *P* < 0.05), but not in evergreen broadleaf species (R^2^ = 0.39, *P* = 0.18) or conifer species (R^2^ = 0.10, *P* = 0.49). We further conducted multiple regression analysis using SMA regression with SRL as the objective variable and SLA and mycorrhizal type as the explanatory variables. The result shows that the slope was not significantly different between the mycorrhizal types (*P* = 0.18). However, comparing their intercept with the common slope (1.63), ECM species had a significantly higher intercept than AM species (*P* < 0.001) and its difference was 0.5 on log_10_ scale (Table 3). A similar shift in the trait–trait relationships occurred when RD or its products (i.e., SRL and SRA) were included (Table S4 and Figure S8a–f available as Supplementary Data at *Tree Physiology* Online). The relationship between RN and LN was positively correlated across the studied species (R^2^ = 0.34, *P* < 0.001) (Table S2 available as Supplementary Data at *Tree Physiology* Online), but the relationship did not shift between mycorrhizal types (Table S5 available as Supplementary Data at *Tree Physiology* Online).

**Table 3:** Intercepts of the relationship between specific root length (SRL) and specific leaf area (SLA) were compared between species symbiotic with arbuscular mycorrhizal fungi (AM species) and species symbiotic with ectomycorrhizal fungi (ECM species) by standard major axis regression using the common slope. The ± in the table indicates variation in the intercept and slope at 95% confidence interval. We used SRL as the objective variable and SLA and symbiotic mycorrhizal fungi type as explanatory variables.

	Slope	Intercept	R^2^
AM species	1.63 ± 0.26	−3.51 ± 1.00	0.47^**^
ECM species		−3.07 ± 0.93	0.88^***^
Significance	*P* = 0.18	*P* < 0.001	

^***^ *P* < 0.001, ^**^P < 0.01.

**Figure 4:**
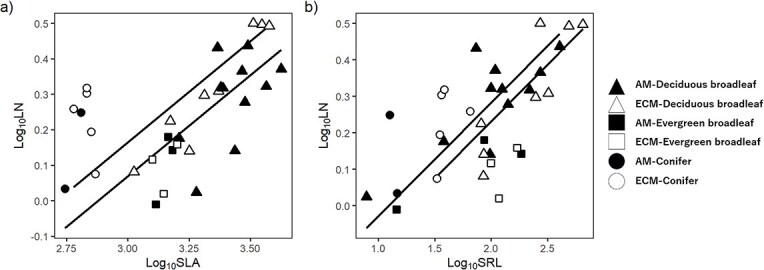
The log–log relationship between LN (%) and SLA (cm^2^ g^−1^) (a) and between LN (%) and SRL (m g^−1^) (b) in mycorrhizal types. Regression lines in mycorrhizal types between SLA and LN are y = 0.57x − 1.64 (R^2^ = 0.37, *P* < 0.01) for AM species and y = 0.57x − 1.55 (R^2^ = 0.32, *P* < 0.05) for ECM species and their intercept was marginally significantly different (*P* = 0.059) with the common slope. Regression lines in mycorrhizal types between SRL and LN are y = 0.31x − 0.39 (R^2^ = 0.51, *P* < 0.01) for species symbiotic with arbuscular mycorrhizal fungi (AM species) and y = 0.31x − 0.34 (R^2^ = 0.35, *P* < 0.05) for species symbiotic with ectomycorrhizal fungi (ECM species) and their intercept was not significantly different (*P* = 0.26) with the common slope.

The relationship between LN and SLA was significantly positive in both AM (R^2^ = 0.37, *P* < 0.01) and ECM species (R^2^ = 0.32, *P* < 0.05) (Figure 4a). There was no significant difference in the slope between AM and ECM species (*P* = 0.97), whereas the difference in the intercept was marginally significant (*P* = 0.059) with the common slope; AM species had consistently lower LN than ECM species at a given SLA. The relationship between LN and LFP also shifted between mycorrhizal types (Figure S8g available as Supplementary Data at *Tree Physiology* Online). The relationship between LN and SRL was positive in both AM species (R^2^ = 0.51, *P* < 0.01) and ECM species (R^2^ = 0.33, *P* < 0.05). Their slope (*P* = 0.36) and intercept (*P* = 0.30) were not significantly different (Figure 4b), indicating that there was no shift in this relationship depending on the two mycorrhizal types.

## Discussion

We hypothesized that root and leaf traits are correlated with each other, but the mycorrhizal types influenced the relationship. Our common garden experiment demonstrated that leaf and root traits and biomass allocation converged on the PES and the positive relationship between SRL and SLA shifted between two mycorrhizal types, supporting our hypothesis. We found that AM species consistently had lower SRL than ECM species when compared at the same SLA, indicating that coordination between root and leaf traits varies between mycorrhizal types.

In both Standard and Phylogenetic PCA, all the studied leaf traits (SLA, LFP and LN) were correlated with PC1, indicating that PC1 represented the LES (Wright et al. 2004). Some root traits (SRL, RTD and RN) were also strongly correlated with PC1 in both Standard PCA and Phylogenetic PCA, indicating that PC1 represented the economic spectrum at the whole-plant level (PES). Thus, on the PES, species with resource acquisitive leaves had resource acquisitive roots, in line with the expectation in earlier studies (Westoby and Wright 2006, Hikosaka and Osone 2009, Freschet et al. 2010, Reich 2014). PC1 and these traits also differed between deciduous and evergreen species, indicating that PES including root trait variations are related to the leaf habits (Hikosaka et al. 2025).

On the other hand, PC2 in both the Standard and Phylogenetic PCA varied between AM and ECM species (Figure S5b and d available as Supplementary Data at *Tree Physiology* Online). Root diameter (RD) is the only trait that was strongly correlated with PC2 more than PC1 in both Standard and Phylogenetic PCA (Table 1;  Table  S4 available as Supplementary Data at *Tree Physiology* Online), and it was significantly different between the mycorrhizal types (Figure 2). These results suggest that mycorrhizal type is an important factor that explains root trait variation as reported in previous studies (Comas et al. 2014, Yan et al. 2022).

**Figure 5:**
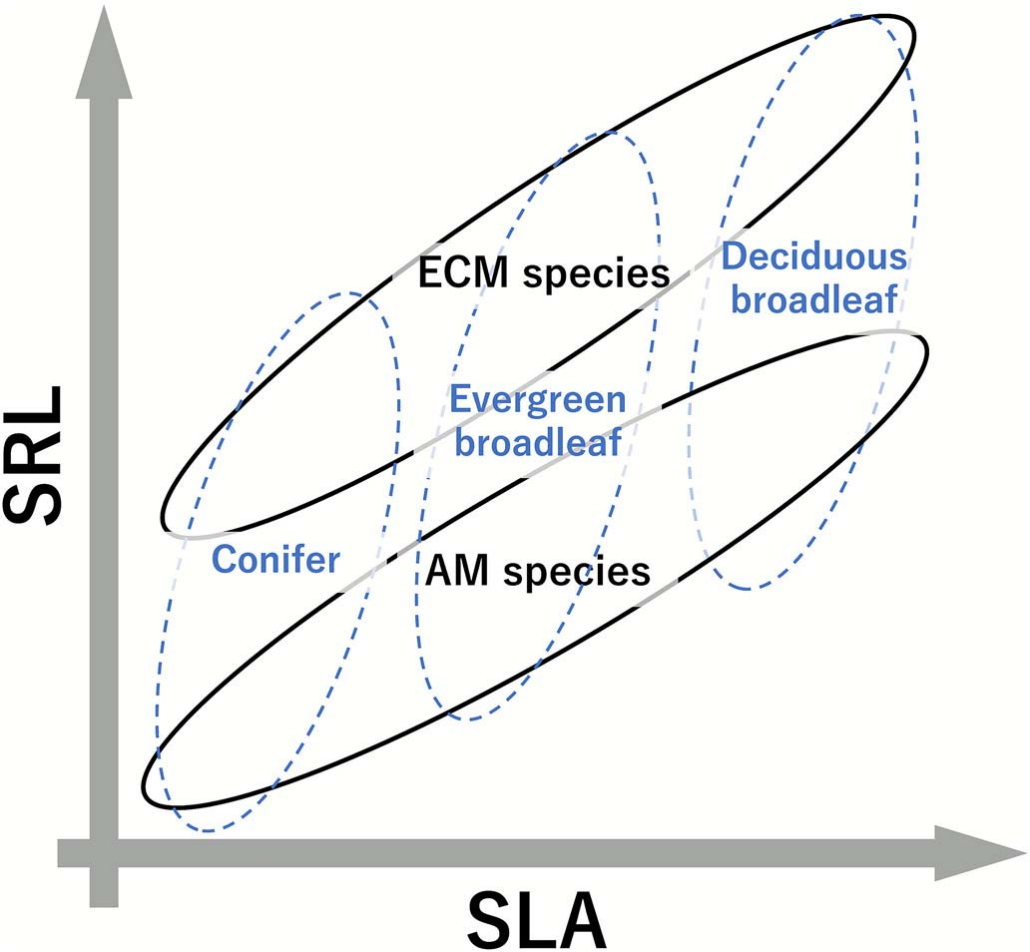
Conceptual diagram for the relationship between specific root length (SRL) and specific leaf area (SLA) across different leaf habits and mycorrhizal types shown in Figure 3. Specific root length (SRL) is coordinated with SLA along the PES. The correlation within leaf habit was weak or non-significant, but it was positive in species symbiotic with arbuscular mycorrhizal fungi (AM species) and species symbiotic with ectomycorrhizal fungi (ECM species). Their intercept of the line differs between the two mycorrhizal types due to the thicker root of AM species. Thus, the relationship between SRL and SLA can become clearer when species are separated by their mycorrhizal types.

We found that the coordination between SRL and SLA shifted between mycorrhizal types (Figure 3), as summarized in a conceptual diagram (Figure 5). This is because SRL is a function of RTD and RD (Eq. (1)). RTD did not differ between mycorrhizal types (Figure S6 available as Supplementary Data at *Tree Physiology* Online) and significantly correlated with SLA as mentioned above. However, AM species had a larger RD than ECM species (Figure 2) because the formers have a thicker root cortex where arbuscular mycorrhizal fungi colonize (Brundrett 2002, Comas et al. 2014, Ma et al. 2018). On the other hand, ECM species do not need a thick root cortex because ectomycorrhizal fungi do not colonize inside the root cell, but on the root surface or root intercellular space (Brundrett 2002, Comas et al. 2014, Ma et al. 2018). Therefore, since RTD represents LES but RD varies depending on the mycorrhizal types, SRL is also influenced and separating species by their mycorrhizal types can make the relationship between SRL and SLA clearer.

One may consider that other factors alter SRL. Because first order roots are thinner than second order roots (McCormack et al. 2015, Liu et al. 2019), ECM species could achieve higher SRL if first order roots were longer than second order roots due to denser branching. However, fraction of first order root length was not different between mycorrhizal types (Figure S6e available as Supplementary Data at *Tree Physiology* Online), suggesting that difference in SRL between AM and ECM species mainly due to RD. Lower SRL has been reported in plants allocating more biomass to roots because lower nutrient uptake efficiency can be offset by higher biomass allocation in roots (Weemstra et al. 2020). In our study, ABR was positively correlated with SRL but not affected by mycorrhizal type, indicating that current-year seedlings of AM trees did not show such compensation. Thus, biomass allocation could not explain the shift in the SRL–SLA relationship. Previous studies also showed that thicker roots are associated with longer root lifespans and greater hyphal production (McCormack et al. 2012, McCormack and Iversen 2019), which can obscure root–leaf trait relationship by altering SRL via Eq. (1). Our study focused only on mycorrhizal type, but considering these additional factors may provide further insights into the functional coordination.

Our finding can explain the discrepancies in the correlation between SRL and SLA amongst earlier studies (Weemstra et al. 2016, Weigelt et al. 2021). These discrepancies are likely due to two factors. First, when the studied species include different mycorrhizal types and are analyzed without separation, the correlation would be weak because of the difference in the intercept between mycorrhizal types (Figure 5). Similar patterns were also observed when RD or its product (i.e., SRL or SRA) was included in the trait–trait relationship analyses (Table S5 and Figure  S8a–f available as Supplementary Data at *Tree Physiology* Online). Hence, the specific types of mycorrhizal symbiosis associated with plant species should be considered in evaluating the correlation between root and leaf morphological traits. Second, the plastic trait variation can explain the discrepancies. We successfully obtained strong correlations between SRL and SLA probably because we minimized the potential intraspecific variation using plants of the same age grown in a common environment. In contrast, root and leaf traits plastically change in response to growth conditions (Weemstra et al. 2017, 2021, Wang et al. 2018), which would obscure the trait–trait relationships in the field where environmental factors are heterogeneous (Freschet et al. 2015). Trait covariation is also influenced by ontogeny (Thomas and Winner 2002, Lusk and Warton 2007, He and Yan 2018, Liu et al. 2020, Garbowski et al. 2021, Leroy et al. 2023) and mycorrhizal infection (Kilpeläinen et al. 2019, Romero et al. 2023). Such environment-, ontogeny- and mycorrhizal infection-dependent intraspecific variations in root and leaf traits may contribute to balancing internal elemental compositions and, in turn, resource acquisition and utilization in respective environments. Although previous ecophysiological studies have revealed the importance of phenotypic plasticity in roots or leaves, their functional coordination under a fluctuating environment and heterogeneous mycorrhizal association remains to be studied.

Interspecific variation in SRL was greater than in SLA within each mycorrhizal type; when the relationship between SRL and SLA was assessed separately for each mycorrhizal type, the common scaling slope was 1.63 (Table 3), indicating that SRL becomes 10-fold when SLA becomes ca. fourfold. This is consistent with a theoretical prediction by Hikosaka and Osone (2009) indicating that larger variation in root traits than in leaf traits is a necessary condition for the positive relationship between LN and photosynthetic rate per mass, which is an important component of the LES. Since carbon concentration in the plant body is nearly constant, LN reflects the nitrogen-to-carbon ratio in the plant body. If nitrogen uptake were constant or scaled equally with carbon gain, LN would not positively correlate with photosynthetic rate, which contradicts LES. Therefore, plants with a higher photosynthetic rate must have a much higher nitrogen uptake rate (Hikosaka and Osone 2009). In contrast to root and leaf morphology, the slope in the RN—LN relationship was 0.68 and RN showed smaller variation than LN, suggesting that coordination between root and leaf chemical trait would not contribute to hold LES. The greater interspecific variation in SRL than that in SLA irrespective of mycorrhizal types would be indispensable for the positive relationship between nitrogen concentration and SLA in LES.

The lower SRL in AM species may result in poorer nutrient uptake ability without mycorrhizal symbiosis. Since we used washed river sand and observed no ECM colonization, we assume that mycorrhizal symbiosis hardly occurred and plants obtained nutrients only via root at least in earlier stages of the growth. This suggests that AM species would be at a disadvantage for nutrient uptake in the absence of symbiosis because of their low root surface area. We did not find significant differences in LN between AM and ECM species at the same SRL (Figure 4b), which indicates that nutrient uptake per unit fine root length or surface area did not differ between the two mycorrhizal types. In contrast, LN was lower in AM than in ECM species at the same SLA (Figure 4a), suggesting that the amount of absorbed nitrogen was relatively smaller for leaf growth in AM species than in ECM species without mycorrhizal symbiosis. In the field, however, AM species might acquire much nutrient through symbiotic mycorrhizal fungi because up to 75% of nitrogen in AM species is delivered from AM (Jin et al. 2005, Tanaka and Yano 2005, Shi et al. 2023); therefore, less root surface in AM species would be compensated by mycorrhizal symbiosis (McCormack and Iversen 2019). Our results imply that SRL is not necessarily a good indicator for nutrient acquisition if mycorrhizal type is ignored.

Some of trait–trait relationships and PCA results changed when phylogeny was considered, probably due to outliers amongst conifer species. For example, trait–trait relationships involving RN or RTD changed when PICs were used and the relationships between PC2 and both RN and RTD differed between the Standard and Phylogenetic PCA, probably because PIC of RN and RTD had outlier at the node conifer. In addition, the relationship between PICs of SLA and SRL was positively correlated when PICs at node *Picea* and conifer were excluded. These would be caused by the differences in evolutionary history between conifers and broadleaf species. Conifers have a higher ratio of the rate of non-synonymous to synonymous nucleotide substitutions (dN/dS) than broadleaf species (De la Torre et al. 2017), indicating greater phenotypic divergence per unit evolutionary distance and stronger diversifying selection after the Last Glaciation (Kujala and Savolainen 2012, De La Torre et al. 2014). When substitution rates differ amongst clades, applying a single evolutionary model across them can lead to misleading results (Garland and Ives 2000, Begum et al. 2021). Therefore, the evolutionary history of conifers, which is away from neutral evolution, may have contributed to outliers in the PICs. When a dataset includes both gymnosperms and angiosperms, the effects of phylogeny on traits may need to be evaluated under different evolutionary scenarios for the two groups. However, our results suggest that the effect of mycorrhizal types on the functional coordination is independent of the evolutionary divergence between conifers and broadleaf species because the shift is caused by RD variation between AM species and ECM species regardless of angiosperms and gymnosperms.

## Conclusion

We found that the root and leaf traits were coordinated with each other across woody species, forming the PES. However, some trait–trait relationships shifted between mycorrhizal types because RD significantly differed between AM and ECM species. In particular, the intercept of the SRL—SLA relationship was different between AM and ECM species. Our results suggest that RD and SRL are not simple economic traits, and we should be careful to discuss their ecological meanings. Our results also suggest that minimizing potential intraspecific variation is effective in detecting the coordination between root and leaf traits across species. The ecological significance of the mycorrhizal effect on traits and functional coordination between root and leaf traits under different environmental conditions remains to be studied.

## Supplementary Material

appendix_tp_R4_1_tpaf151_cleaned

## Data Availability

The dataset is available on supporting information (Table S1 available as Supplementary data at *Tree Physiology* Online).
