# Supplementary material for: The coordination between root and leaf functional traits across 33 woody plant species shifts between mycorrhizal types
Source: Tree Physiol. 2025 Dec 10;46(1):tpaf151. doi: 10.1093/treephys/tpaf151 (PMC12828701; doi:10.1093/treephys/tpaf151)
Supplement: appendix_tp_R4_1_tpaf151_cleaned [file appendix_tp_r4_1_tpaf151_cleaned.docx]

Tree physiology

Research Papers

The coordination between root and leaf functional traits across 33 woody plant species shifts between mycorrhizal types

Katsumi C. Suzuki^1*^, Hirofumi Kajino^1^, Shusaku Hirokawa^1^, Hajime Tomimatsu^1^, Kohmei Kadowaki^2,3^, Kouki Hikosaka^1*^

^1^Graduate School of Life Science, Tohoku University, Aramakiaza Aoba 6-3, Aoba-ku, Sendai, 980-8578, Japan

^2^The Hakubi Center for Advanced Research, Kyoto University, Kyoto, Japan

^3^Graduate School of Agriculture, Kyoto University, Kyoto, Japan

*Corresponding author

Katsumi C. Suzuki (katumi.suzuki.p8@dc.tohoku.ac.jp)

Kouki Hikosaka (hikosaka@tohoku.ac.jp)

Supporting information

**Table S1** List of the species and trait values. The mycorrhizal types of each species were classified based on Maherali et al. (2016) and Soudzilovskaia et al. (2020) and growth form was classified based on Hayashi (2014). RD, root diameter; SRL, specific root length; SRA, specific root area; RTD, root tissue density; RN, root nitrogen concentration; STD, stem tissue density; ABR, Above- and Below-ground ratio; SLA, specific leaf area; LFP, leaf force to punch; LN, leaf nitrogen concentration;AM, arbuscular mycorrhizal fungi; ECM; ectomycorrhizal species; ERM, ericoid mycorrhizal fungi

| Name | Mycorrhizal type | Leaf habit | Growth form | RD  (mm) | SRL  (m g^-1^) | SRA  (mm^2^ mg^-1^) | RTD  (mg mm^-3^) | RN  (%) | STD  (g cm^-3^) | ABR | LFP  (N mm^-1^) | SLA  (cm^2^ g^-1^) | LN  (%) | Fraction of 1st order root length (mm mm^-1^) |
| --- | --- | --- | --- | --- | --- | --- | --- | --- | --- | --- | --- | --- | --- | --- |
| *Abies firma* | ECM | Evergreen Conifer | Tree | 0.462 | 33.0 | 60.7 | 0.106 | 1.48 | 0.505 | 1.10 | 0.622 | 737 | 1.19 | 0.473 |
| *Abies sachalinensis* | ECM | Evergreen Conifer | Tree | 0.283 | 36.4 | 41.1 | 0.253 | 1.64 | 0.314 | 1.06 | 0.487 | 680 | 2.01 | 0.426 |
| *Acer palmatum* | AM | Deciduous broadleaf | Tree | 0.248 | 141.1 | 117.5 | 0.118 | 1.71 | 0.242 | 1.62 | 0.145 | 3003 | 1.89 | 0.193 |
| *Betula ermanii* | ECM | Deciduous broadleaf | Tree | 0.244 | 272.0 | 278.0 | 0.041 | 2.74 | 0.582 | 2.24 | 0.082 | 3249 | 3.16 | 0.287 |
| *Betula maximowicziana* | ECM | Deciduous broadleaf | Tree | 0.204 | 322.1 | 208.9 | 0.083 | 1.48 | 0.331 | 1.54 | 0.161 | 2347 | 2.04 | 0.244 |
| *Betula platyphylla* | ECM | Deciduous broadleaf | Tree | 0.205 | 649.7 | 462.0 | 0.034 | 2.05 | 0.210 | 2.12 | 0.103 | 3524 | 3.14 | 0.303 |
| *Betura grossa* | ECM | Deciduous broadleaf | Tree | 0.202 | 490.1 | 376.4 | 0.038 | 2.32 | 0.256 | 1.55 | 0.086 | 3772 | 3.10 | 0.289 |
| *Callicarpa japonica* | AM | Deciduous broadleaf | Shrub | 0.196 | 272.9 | 198.1 | 0.074 | 1.59 | 0.128 | 2.25 | 0.138 | 2925 | 2.32 | 0.130 |
| *Carpinus tschonoskii* | ECM | Deciduous broadleaf | Tree | 0.190 | 248.6 | 163.7 | 0.111 | 2.09 | 0.257 | 1.65 | 0.234 | 2051 | 1.98 | 0.262 |
| *Cephalotaxus harringtonia* | AM | Evergreen Conifer | Tree | 0.752 | 14.8 | 39.3 | 0.117 | 1.50 | 0.209 | 2.96 | 0.557 | 555 | 1.08 | 0.645 |
| *Cercidiphyllum japonicum* | AM | Deciduous broadleaf | Tree | 0.310 | 99.8 | 106.7 | 0.111 | 1.27 | 0.384 | 1.50 | 0.125 | 3668 | 2.10 | 0.213 |
| *Clethra barbinervis* | AM | Deciduous broadleaf | Tree | 0.327 | 108.7 | 128.9 | 0.101 | 1.26 | 0.168 | 1.15 | 0.150 | 4220 | 2.35 | 0.371 |
| *Cleyera japonica* | AM | Evergreen broadleaf | Tree | 0.295 | 185.2 | 224.7 | 0.062 | 1.78 | 0.359 | 1.69 | 0.446 | 1520 | 1.39 | 0.278 |
| *Cornus kousa* | AM | Deciduous broadleaf | Tree | 0.572 | 97.6 | 193.0 | 0.032 | 1.04 | 0.161 | 1.36 | 0.208 | 2733 | 1.38 | 0.287 |
| *Cornus macrophylla* | AM | Deciduous broadleaf | Tree | 0.481 | 125.1 | 210.5 | 0.070 | 1.26 | 0.253 | 2.05 | 0.259 | 2393 | 2.09 | 0.419 |
| *Cryptomeria japonica* | AM | Evergreen Conifer | Tree | 0.425 | 12.7 | 18.5 | 0.475 | 1.46 | 0.184 | 1.08 | 0.257 | 644 | 1.77 | 0.569 |
| *Fagus crenata* | ECM | Deciduous broadleaf | Tree | 0.147 | 86.2 | 54.5 | 0.345 | 1.48 | 0.269 | 1.03 | 0.391 | 1783 | 1.38 | 0.097 |
| *Fraxinus sieboldiana* | AM | Deciduous broadleaf | Tree | 0.489 | 38.0 | 73.2 | 0.080 | 1.31 | 0.259 | 0.45 | 0.402 | 1617 | 1.50 | 0.395 |
| *Ilex crenata* | AM | Evergreen broadleaf | Shrub | 0.356 | 86.5 | 105.3 | 0.094 | 1.27 | 0.145 | 1.61 | 0.422 | 1459 | 1.51 | 0.325 |
| *Lindera erythrocarpa* | AM | Deciduous broadleaf | Tree | 0.891 | 7.9 | 23.7 | 0.164 | 1.18 | 0.241 | 0.97 | 0.187 | 1903 | 1.06 | 0.172 |
| *Mallotus japonicus* | AM | Deciduous broadleaf | Tree | 0.426 | 73.4 | 118.8 | 0.062 | 1.91 | 0.203 | 1.40 | 0.172 | 2323 | 2.70 | 0.254 |
| *Morus australis* | AM | Deciduous broadleaf | Tree | 0.324 | 405.2 | 475.9 | 0.021 | 1.66 | 0.171 | 1.44 | 0.088 | 3086 | 2.73 | 0.217 |
| *Neolitsea sericea* | AM | Evergreen broadleaf | Tree | 0.838 | 14.5 | 42.5 | 0.095 | 1.80 | 0.245 | 0.93 | 0.717 | 1302 | 0.98 | 0.685 |
| *Picea glehnii* | ECM | Evergreen Conifer | Tree | 0.507 | 43.9 | 60.9 | 0.086 | 1.79 | 1.834 | 1.15 | 0.233 | 602 | 1.81 | 0.598 |
| *Picea jezoensis* | ECM | Evergreen Conifer | Tree | 0.535 | 38.6 | 81.4 | 0.068 | 1.56 | 0.533 | 0.73 | 0.235 | 681 | 2.08 | 0.369 |
| *Pieris japonica* | ERM | Evergreen broadleaf | Shrub | 0.115 | 384.8 | 158.5 | 0.151 | 1.28 | 0.254 | 3.51 | 0.317 | 1850 | 1.55 | 0.099 |
| *Pinus densiflora* | ECM | Evergreen Conifer | Tree | 0.359 | 35.1 | 58.8 | 0.109 | 1.38 | 0.435 | 1.14 | 0.273 | 709 | 1.57 | 0.471 |
| *Quercus acuta* | ECM | Evergreen broadleaf | Tree | 0.216 | 99.5 | 101.8 | 0.094 | 1.17 | 0.293 | 1.14 | 0.978 | 1259 | 1.31 | 0.201 |
| *Quercus crispula* | ECM | Deciduous broadleaf | Tree | 0.214 | 81.7 | 85.4 | 0.093 | 1.05 | 0.348 | 0.32 | 0.602 | 1491 | 1.68 | 0.131 |
| *Quercus glauca* | ECM | Evergreen broadleaf | Tree | 0.241 | 117.1 | 126.8 | 0.085 | 1.17 | 0.289 | 1.12 | 0.911 | 1400 | 1.05 | 0.214 |
| *Quercus serrata* | ECM | Evergreen broadleaf | Tree | 0.155 | 170.3 | 98.7 | 0.163 | 1.29 | 0.317 | 0.35 | 0.579 | 1588 | 1.44 | 0.165 |
| *Quercus salicina* | ECM | Deciduous broadleaf | Tree | 0.242 | 85.5 | 94.8 | 0.089 | 1.24 | 0.291 | 1.09 | 1.193 | 1060 | 1.21 | 0.077 |
| *Sorbus commixta* | AM | Deciduous broadleaf | Tree | 0.223 | 219.1 | 166.4 | 0.093 | 1.76 | 0.259 | 1.10 | 0.118 | 2442 | 2.08 | 0.222 |

**Table S2** Pagel’s λ and coefficient of variation (CV) of each the studied traits. Pagel’s λ ranges from 0 to 1, and higher value indicates stronger phylogenetic signal. Pagel’s λ was calculated based on phylogenetic tree (Fig.S1). RD, root diameter; SRL, specific root length; SRA, specific root area; RTD, root tissue density; RN, root nitrogen concentration; STD, stem tissue density; ABR, Above- and Below-ground ratio; SLA, specific leaf area; LFP, leaf force to punch; LN, leaf nitrogen concentration

| Trait | Pagel‘s λ | CV (%) |
| --- | --- | --- |
| **RD** | 0.92*** | 55.1 |
| **SRL** | 0.96*** | 98.1 |
| **SRA** | 0.92*** | 77.0 |
| **RTD** | 0.61 | 80.1 |
| **RN** | 0.80* | 24.4 |
| **STD** | 0.55 | 87.6 |
| **ABR** | 0.39 | 48.4 |
| **SLA** | 0.94*** | 77.7 |
| **LFP** | 0.88*** | 53.3 |
| **LN** | 0.82* | 33.6 |
| **Fraction of 1st order root length** | 0.53*** | 52.2 |

***p < 0.001, **p < 0.01, *p < 0.05

**Table S3** R^2^ in the relationship between SRL and SLA in each mycorrhizal type without conifers. AM, species symbiotic with arbuscular mycorrhizal fungi; ECM species, species symbiotic with ectomycorrhizal fungi

|  | R^2^ |
| --- | --- |
| Species without conifers | 0.21* |
| AM species without conifers | 0.26† |
| ECM species without conifers | 0.81*** |

****P* < 0.001, ***P* < 0.01, **P* < 0.05, †*P* < 0.1

**Table S4** The effects of mycorrhizal types on the trait-trait relationship. Upper right of this table shows the significance of difference in slope between AM species and ECM species (black) and lower left of this table shows the significance of difference in intercept between AM species and ECM species (red). The cell was highlighted when the trait-trait correlation in each mycorrhizal type was significant, and bold letters indicated that either intercept or slope was significantly different between AM species and ECM species. Under line shows that both trait-trait correlation in two mycorrhizal type and difference between mycorrhizal types were significant (*P* < 0.1). RD, root diameter; SRL, specific root length; SRA, specific root area; RTD, root tissue density; RN, root nitrogen concentration; STD, stem tissue density; ABR, Above- and Below-ground ratio; SLA, specific leaf area; LFP, leaf force to punch; LN, leaf nitrogen concentration

|  | **RD** | **SRL** | **SRA** | **RTD** | **RN** | **STD** | **ABR** | **SLA** | **LFP** | **LN** |
| --- | --- | --- | --- | --- | --- | --- | --- | --- | --- | --- |
| **RD** |  | **ns** | **ns** | ns | ns | ***** | **ns** | **ns** | **ns** | **ns** |
| **SRL** | ***** |  | **ns** | **ns** | **†** | ***** | ns | **ns** | **†** | ns |
| **SRA** | ***** | **†** |  | ns | **†** | **†** | **ns** | **ns** | **†** | ns |
| **RTD** | **†** | **†** | ns |  | ns | **ns** | ns | ns | ns | ns |
| **RN** | ns | **ns** | **ns** | ns |  | **ns** | **ns** | **ns** | ns | ns |
| **STD** | ******* | ******* | ****** | ***** | **†** |  | ns | **ns** | ns | **ns** |
| **ABR** | ******* | ***** | **†** | ns | ***** | ns |  | **ns** | **ns** | **ns** |
| **SLA** | ******* | ******* | ****** | ns | **†** | **†** | **†** |  | ns | **ns** |
| **LFP** | ******* | ****** | **†** | ns | ***** | ns | **†** | ns |  | **ns** |
| **LN** | ***** | ns | ns | ns | ns | ****** | **†** | **†** | ****** |  |

****P* < 0.001, ***P* < 0.01, **P* < 0.05. †*P* < 0.1


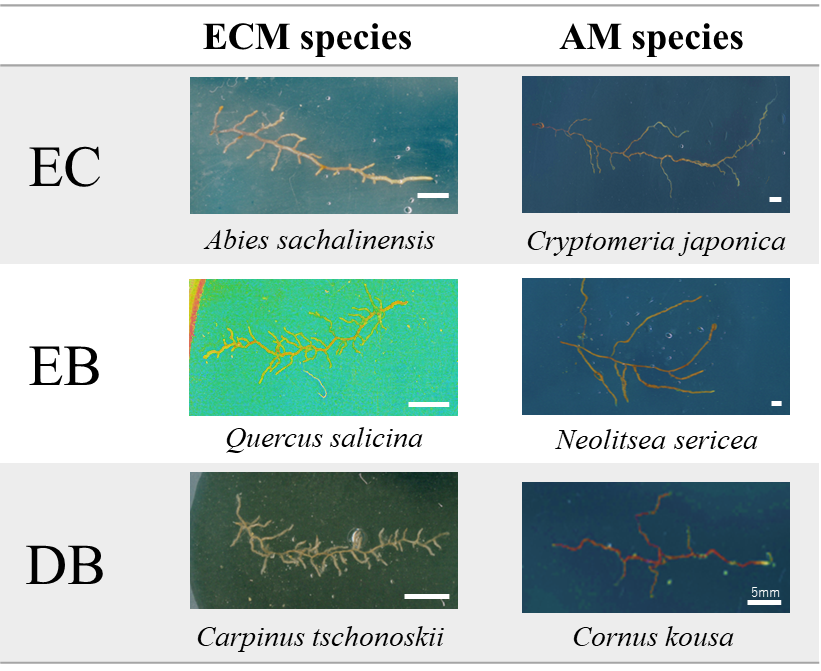


**Fig. S1** Root of each leaf habits (DB, deciduous broadleaf species; EB, evergreen broadleaf species; EC, evergreen conifer species) and mycorrhizal types (AM, species symbiotic with arbuscular mycorrhizal fungi; ECM, species symbiotic with ectomycorrhizal fungi). White bar is a 5 mm scale bar, and color was changed to make it easier to see.


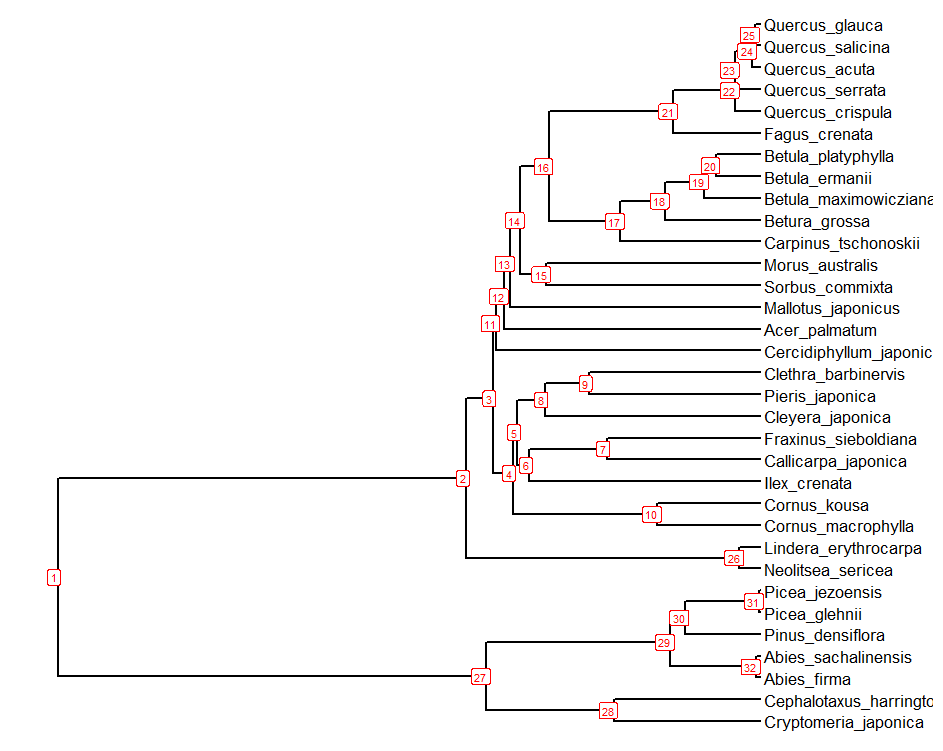


**Fig. S2** The phylogenetic tree of the studied species. Red number indicates node number. We calculated PICs values at all internal nodes (1-32), and we used every PICs value in evaluating the correlation between PICs of traits at all internal nodes (1-32) according to Harvey and Pagel (1991).

**
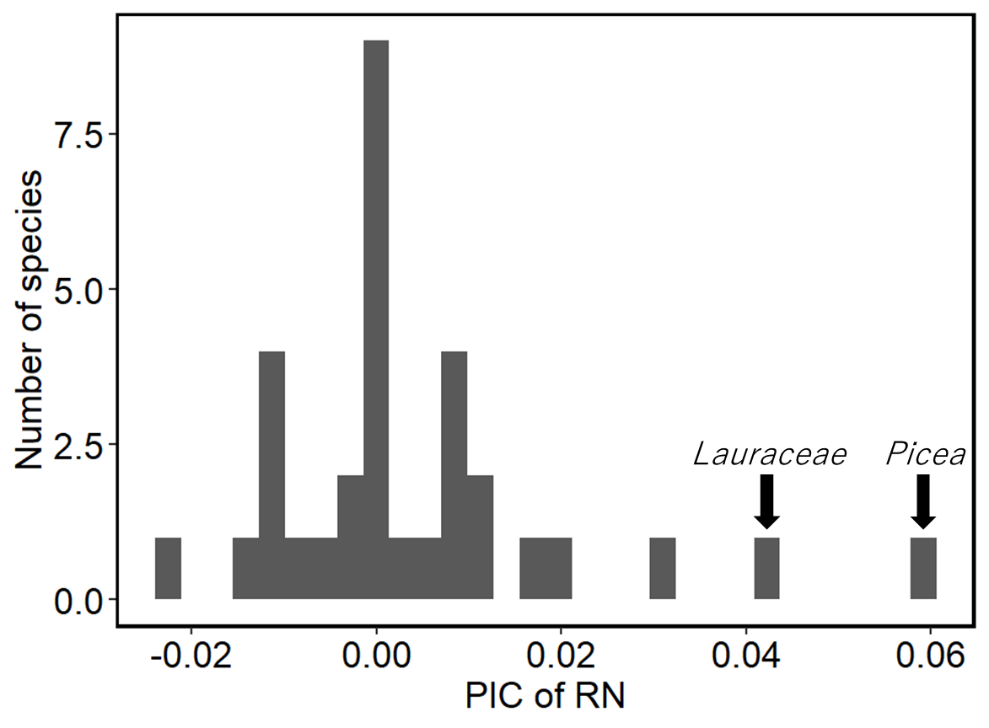
**

**Fig. S3** Histogram of phylogenetic independent contrast (PIC) of root nitrogen concentration (RN, %). PICs of RN were highest at node *Picea* (node 31 in Fig. S2) and second highest at node *Lauraceae* (node 26 in Fig. S2)

**
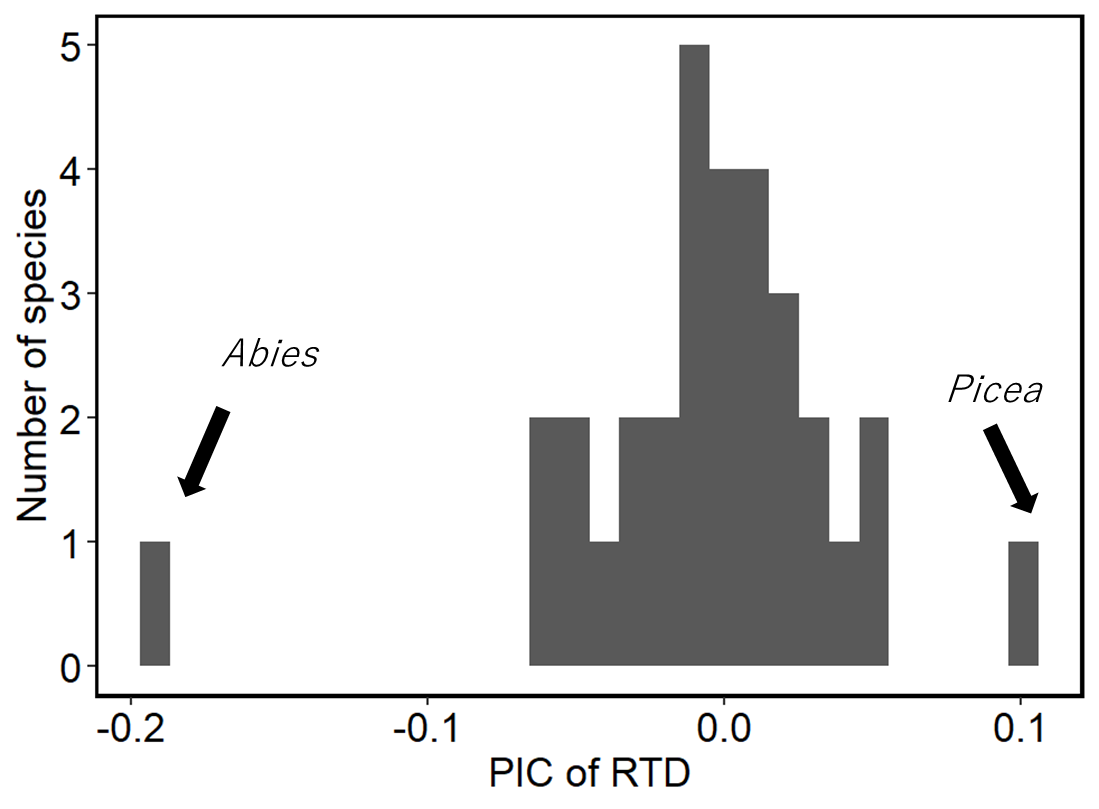
**

**Fig. S4** Histogram of phylogenetic independent contrast (PIC) of root tissue density (RTD, mg mm^-3^). PICs of RTD were highest at node *Picea* (node 31 in Fig. S2) and node *Abies* (node 32 in Fig. S2).

**
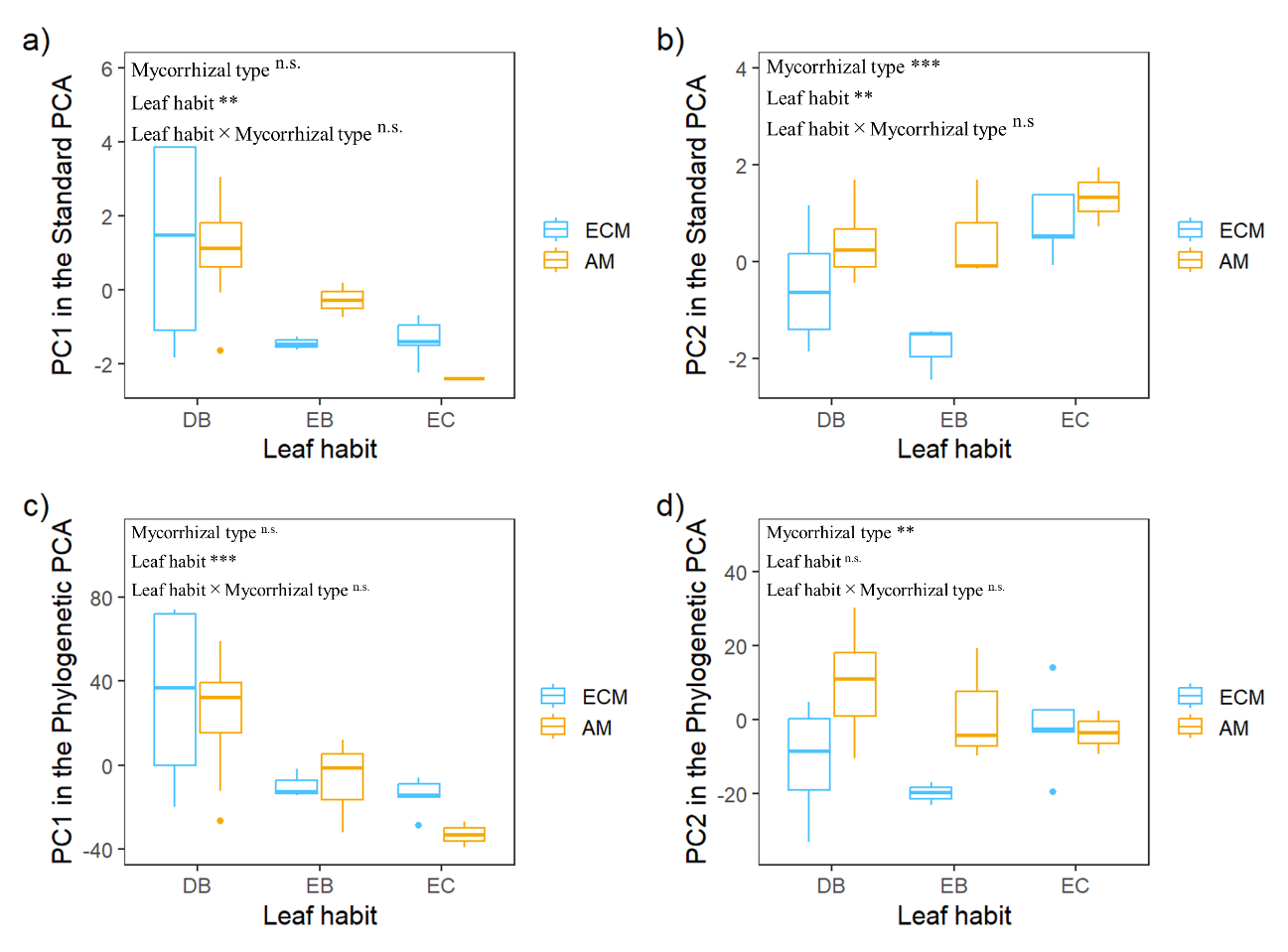
**

**Fig. S5** Comparison of the PC1 (**a**) and PC2 (**b**) scores in the Standard PCA and the PC1 (**c**) and PC2 (d) scores in the Phylogenetic PCA among leaf habits (DB, deciduous broadleaf species; EB, evergreen broadleaf species; EC, evergreen conifer species) and mycorrhizal types (AM, species symbiotic with arbuscular mycorrhizal fungi; ECM, species symbiotic with ectomycorrhizal fungi). We conducted two-way ANOVA using PC1 and PC2 score in the standard PCA as the objective variable and leaf habits and mycorrhizal type as the explanatory variables, and result of two-way ANOVA is shown in upper (**P* < 0.05, ***P* < 0.01, ****P* < 0.001). The effects are listed in order: mycorrhizal type (main effect), leaf habit (main effect), and leaf habit × mycorrhizal type (interaction).

**
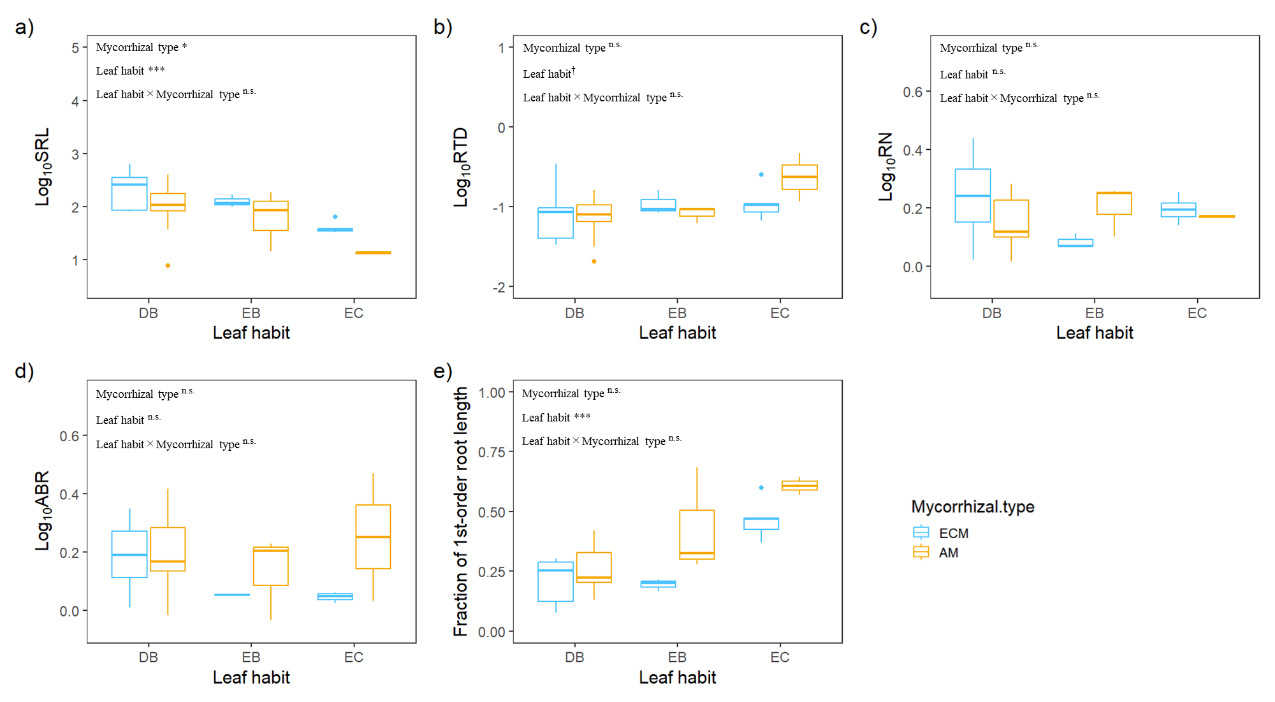
**

**Fig. S6** Specific root length (SRL, m g^-1^) (**a**), root tissue density (RTD, mg mm^-3^) (**b**), root nitrogen concentration (RN, ％) (**c**), above- to below-ground mass ratio (ABR, g g^-1^) (**d**), and fraction of 1st-order root length in total fine root length (**e**) (Fraction of 1^st^-order root length, mm mm^-1^) were compared among leaf habits (DB, deciduous broadleaf species; EB, evergreen broadleaf species; EC, evergreen conifer species) and mycorrhizal types (AM, species symbiotic with arbuscular mycorrhizal fungi; ECM, species symbiotic with ectomycorrhizal fungi). The result of two-way ANOVA is shown in upper (****P* < 0.001, ***P* < 0.01, **P* < 0.05. †*P* < 0.1), and the effects are listed in order: mycorrhizal type (main effect), leaf habit (main effect), and leaf habit × mycorrhizal type (interaction)


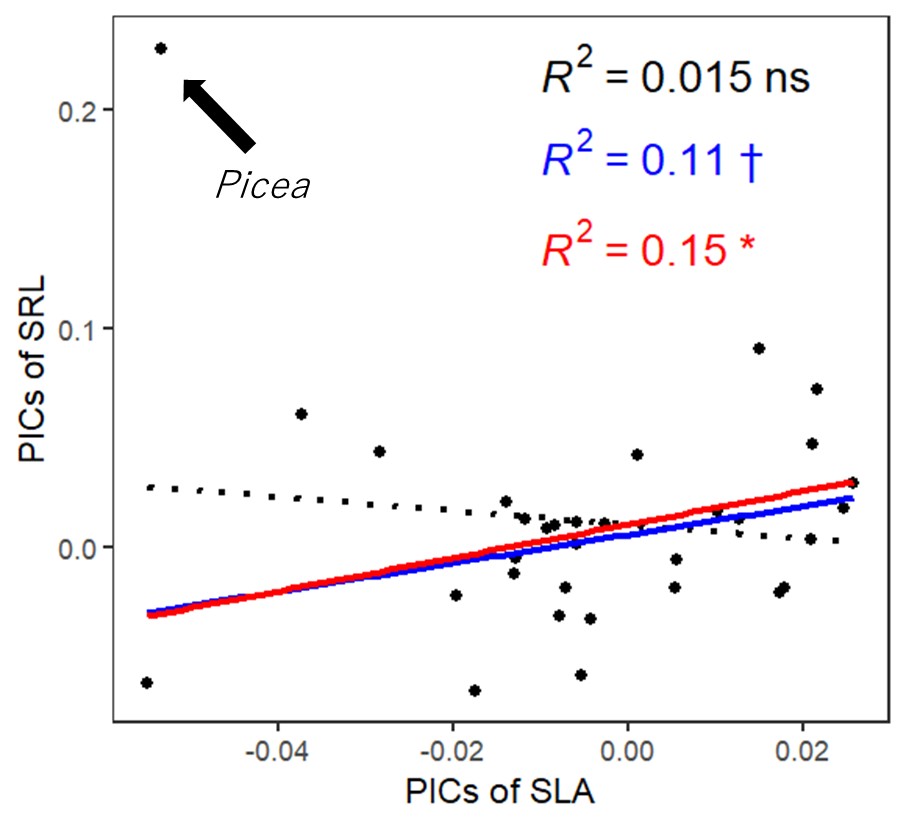


**Fig. S7** The relationship between phylogenetic independent contrast (PIC) of specific root length (SRL, m g^-1^) and specific leaf area (SLA, cm^2^ g^-1^). The black and dotted regression line and R^2^ was calculated using all PICs values, the blue regression line and R^2^ was calculated without PICs value at node *Picea* (node 31 in Fig. S2), and the red regression line and R^2^ was calculated without PICs value at nodes conifer (node 27-32 in Fig. S2).When PICs value at node *Picea* was excluded, the relationship between PICs of SRL and SLA was marginally significant (*P* = 0.06). When PICs value at node conifer was excluded, the relationship between PICs of SRL and SLA was significantly positive (*P* < 0.01).


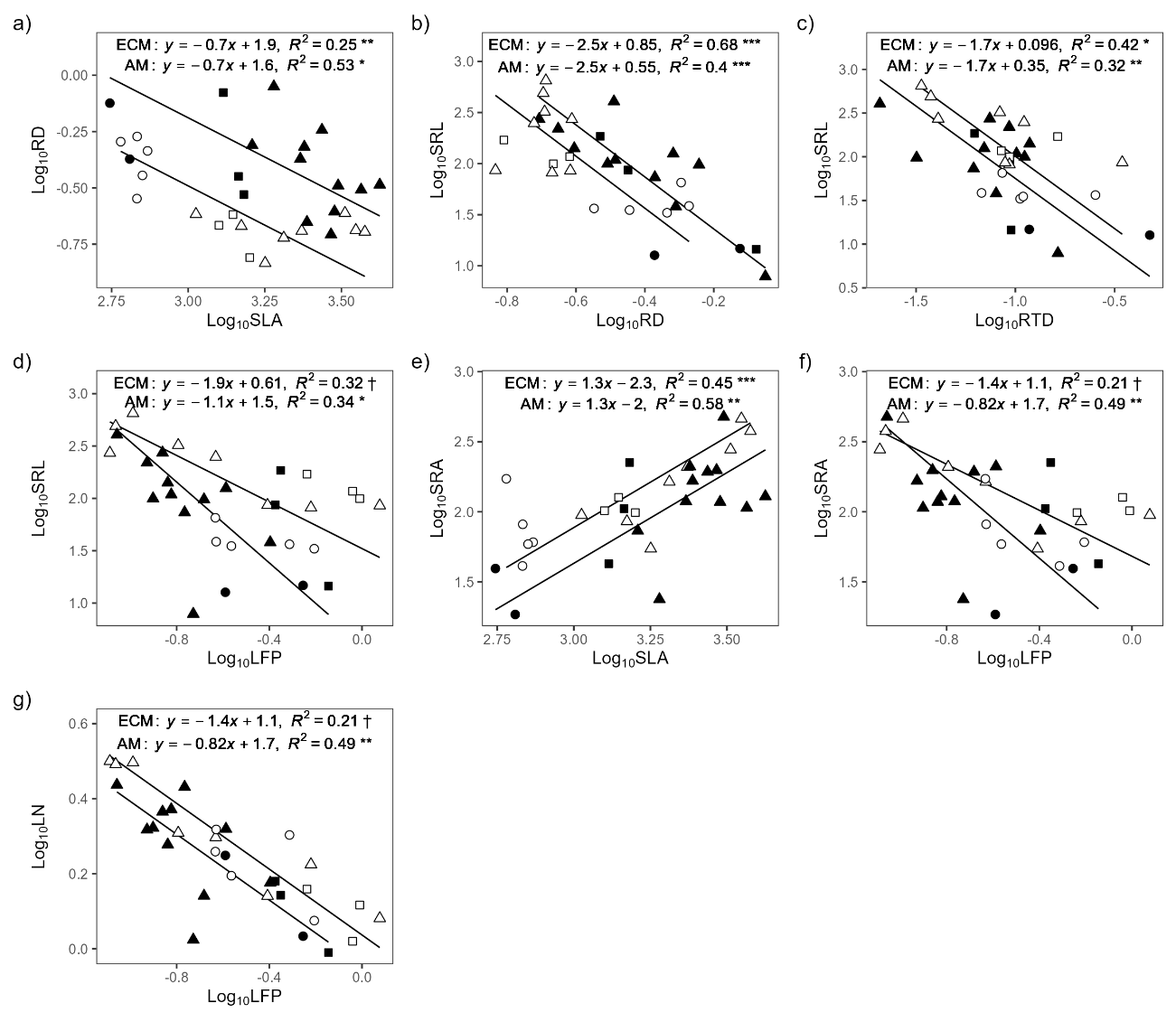


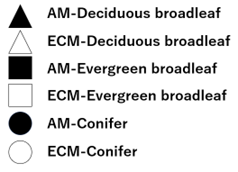


**Fig. S8** The log-log relationship between RD (mm) and SLA (cm^2^ g^-1^) (**a**), between SRL (m g^-1^) and RD (mm) (**b**), between SRL (m g^-1^) and RTD (cm^3^ g^-1^) (**c**), between SRL (m g^-1^) and LFP (N mm^-1^) (**d**), between SRA (cm^2^ g^-1^) and SLA (cm^2^ g^-1^) (**e**), between SRA (cm^2^ g^-1^) and LFP (N mm^-1^) (**f**), and between LN (%) and LFP (N mm^-1^) (**g**) in mycorrhizal type. When the slope was not significantly different (Table S5), a regression line was applied using common slope. Equation of regression line, R^2^, and significance (****P* < 0.001, ***P* < 0.01, **P* < 0.05. †*P* < 0.1) in two mycorrhizal types were also shown.
